# Identification of a defined linear epitope in the OspA protein of the Lyme disease spirochetes that elicits bactericidal antibody responses: Implications for vaccine development

**DOI:** 10.1016/j.vaccine.2017.04.079

**Published:** 2017-05-04

**Authors:** Jerilyn R. Izac, Lee D. Oliver, Christopher G. Earnhart, Richard T. Marconi

**Affiliations:** Dept. Microbiology and Immunology, Virginia Commonwealth University Medical Center, Richmond, VA, United States

**Keywords:** Lyme disease, Lyme vaccine, Outer surface protein A, VANGUARDcrLyme, Chimeritope, OspC, Ixodes ticks

## Abstract

The lipoprotein OspA is produced by the Lyme disease spirochetes primarily in unfed ticks. OspA production is down-regulated by the blood meal and it is not produced in mammals except for possible transient production during late stage infection in patients with Lyme arthritis. Vaccination with OspA elicits antibody (Ab) that can target spirochetes in the tick midgut during feeding and inhibit transmission to mammals. OspA was the primary component of the human LYMErix^™^ vaccine. LYMErix^™^ was available from 1998 to 2002 but then pulled from the market due to declining sales as a result of unsubstantiated concerns about vaccination induced adverse events and poor efficacy. It was postulated that a segment of OspA that shares sequence similarity with a region in human LFA-1 and may trigger putative autoimmune events. While evidence supporting such a link has not been demonstrated, most efforts to move forward with OspA as a vaccine component have sought to eliminate this region of concern. Here we identify an OspA linear epitope localized within OspA amino acid residues 221–240 (OspA_221–240_) that lacks the OspA region suggested to elicit autoimmunity. A peptide consisting of residues 221–240 was immunogenic in mice. Ab raised against OspA_221–240_ peptide surface labeled *B. burgdorferi* in IFAs and displayed potent Ab mediated-complement dependent bactericidal activity. BLAST analyses identified several variants of OspA_221–240_ and a closely related sequence in OspB. It is our hypothesis that integration of the OspA_221–240_ epitope into a multivalent-OspC based chimeric epitope based vaccine antigen (chimeritope) could result in a subunit vaccine that protects against Lyme disease through synergistic mechanisms.

## 1. Introduction

Lyme disease, the most common tick-borne disease in the Northern Hemisphere, occurs in North America, Europe and Asia. The CDC estimates that there are at least 300,000 cases of human Lyme disease per year in the United States (http://www.cdc.gov). Lyme disease is also a significant health concern in veterinary medicine particularly in canines and equines [1,2]. In 2015, over 250,000 positive canine Lyme disease Ab tests were reported to the Companion Animal Parasite Council (CAPC; http://www.capcvet.org/). Since only ~30% of test data are compiled, the actual number of Ab positive tests in canines is certainly much greater than 250,000 and may exceed 800,000. The incidence of Lyme disease in equines is less clear as only a few studies have investigated this. Seropositivity rates in equines in some regions of the United States are as high as 20–35% [3,4] (Marconi, RT; unpublished data). Equine Lyme disease has been reported in N. America, S. America, Asia and Europe [5–8].

The absence of reliable diagnostic assays for early Lyme disease makes it difficult to obtain confirmative serology to support what is largely a clinical diagnosis. This coupled with ongoing debate about appropriate treatment strategies and the potential for debilitating late stage disease outcomes, prevention of Lyme disease through vaccination offers a cost effective approach for prevention. While there are several Lyme disease vaccines labeled for use in canines, vaccines labeled for use in humans or equines are not commercially available. LYMErix^™^, a lipidated Osp (outer surface protein) A based vaccine, was available for human use from 1998 to 2002 before being withdrawn from the market [9,10]. OspA is a linear plasmid (linear plasmid of 54 kb; lp54) encoded lipoprotein that is co-transcribed with OspB [11]. OspA and OspB production are regulated by environmental conditions and selectively produced by spirochetes in the midgut of unfed ticks [12–16]. When *Ixodes* ticks feed, *ospAB* transcription is halted [17–19] and there is no significant production of OspA and B in mammals. A possible exception is transient production in humans suffering from late stage Lyme arthritis [20,21]. While OspAB do not typically elicit Ab responses during natural infections, Ab elicited by vaccination with OspAB can elicit varying levels of protection. Anti-OspAB Ab can target spirochetes in the tick midgut during the blood meal and thereby inhibit transmission from ticks to mammals [22]. As expected for a transmission blocking vaccine that specifically targets a pathogen in its arthropod vector, protection is strictly dependent on circulating Ab titer. This dependency most likely explains the relatively low efficacy (73% efficacy) observed with LYMErix^™^ even after a three dose schedule delivered over 18 months (reviewed in [23]).

Recombinant non-lipidated OspA is also a core component of two subunit canine Lyme disease vaccines. VANGUARD^®^crLYME (ZoetisUS) consists of r-OspA and a unique r-OspC derived chimeric epitope based protein (chimeritope) [24–27] and RECOMBITEX^®^ Lyme (Merial) consists of OspA alone. The limitations of OspA (discussed above) suggest that it alone is insufficient to effectively serve as primary component of future vaccine formulations [9,10,24,28–32]. In addition, earlier studies postulated that OspA residues 165–173 (OspA_165–173_; IYVIEGTSKQDLTSF) elicit Ab that could be cross-reactive with human LFA-1 protein triggering vaccine induced arthritis and autoimmune reactions [33,34]. Several research groups have sought to generate modified OspA proteins or OspA chimerics that lack the epitope of putative concern [33,35–37]. In this study we sought to identify a means by which the positive protective effects of OspA can be exploited in the context of an epitope based chimeric protein. By incorporating an isolated OspA epitope into an OspC epitope based vaccine [25–27,38], hypothetical concerns about adverse events associated with the use of full length OspA can be eliminated.

As alluded to above, we have pursued the development of chimeric epitope based protein vaccines (chimeritopes) for tick-borne diseases [25–27]. Chimeritopes offer advantages over traditional subunit vaccines and protein chimeric vaccines. They can be designed to include a diverse array of linear epitopes derived from multiple variants of a protein and thus provide broad protective efficacy. Importantly, regions of a protein putatively associated with adverse events, such as OspA_165–173_ region, can be omitted. The OspC based chimeritope included in the VANGUARD^®^crLyme canine vaccine consists of a series of linear epitopes (designated as the L5 and H5 epitopes) derived from multiple OspC types [25–27,39]. OspC is an attractive candidate for vaccine development because of it antigenic properties and expression patterns. OspC production is significantly upregulated by exposure to blood in ticks [15,40] and expression remains high during early stage infection in mammals [41]. The primary goal of this study was to identify a defined linear epitope of OspA for potential inclusion into an OspC chimeritope. A combined OspC/OspA chimeritope has the potential to convey protection through independent but potentially synergistic mechanisms: (1) Ab targeting of spirochetes in ticks to inhibit transmission [42,43], and (2) killing of spirochetes in mammals by α-OspC Ab [44]. In summary, an OspA linear epitope spanning residues 221–240 (OspA_221–240_) was identified. Peptide corresponding to this region elicited Ab in mice that surface labeled *B. burgdorferi* and killed in an Ab mediated-complement dependent manner. This study supports the possible inclusion of the OspA_221–240_ epitope variants in an OspC based chimeritope.

## 2. Materials and methods

### 2.1. Bacterial strains and bacterial cultivation

*Borrelia* isolates employed in the study are described in Table 1. All isolates were cultivated in BSK-H complete medium (Sigma-Aldrich) supplemented with 6% rabbit serum (37 °C, 5% CO_2_). Growth was monitored by dark-field microscopy.

### 2.2. Protein production and purification

Recombinant full-length OspA (minus the leader peptide) and OspA subfragments (~50 aa subfragments with 25 aa overlaps) were generated by PCR amplification using *B. burgdorferi* B31 DNA as template [11] (GenBank: CAA32579.1). All PCR primers were deigned with tail sequences to facilitate ligase independent cloning (LIC) and annealing into pET-32 Ek/LIC (Novagen). Proteins produced with this vector possess an N-terminal S-Tag. All primer sequences are listed in Table 2 with the segment of the protein encoded by each amplicon indicated. PCR was performed using standard cycling conditions and *Pfu* polymerase (Promega). Amplicons were purified using PCR purification kits (Qiagen) and then annealed with linearized pET-32 Ek/LIC per the supplier’s protocol (Novagen). The plasmids were propagated in *E. coli* NovaBlue (DE3) cells (Novagen), recovered using QiaFilter midi-plasmid purification kits (QIAGEN) and the inserts sequenced on a fee for service basis (MWG Biotech). For protein production, *E. coli* BL21 (DE3) cells were transformed with the recombinant pET-32 Ek/LIC plasmids and protein expression induced with 1 mM isopropyl-beta-D-thiogalactopyranoside (IPTG) using standard protocols. Cells were harvested and the proteins purified by nickel affinity chro- matography. The recombinant proteins were dialyzed across a 10 kDa molecular weight cutoff membrane (Slide-a-Lyzer, Pierce) against phosphate buffered saline (PBS; pH 7.4). The soluble protein concentration was determined using the bicinchoninic acid assay (BCA; Pierce) and purity assessed by SDS-PAGE as detailed below.

### 2.3. SDS-PAGE, immunoblotting and epitope mapping analyses

Full-length OspA and ~50 aa subfragments (with 25 aa overlaps) were fractionated in 12.5% Criterion Precast Gels (Bio-Rad) and transferred to PVDF membranes using a Transblot Turbo System (Biorad). The membranes were incubated with blocking solution (1X PBS, 0.2% Tween 20, 5% non-fat dry milk) to prevent non-specific binding and screened with sera from C3H/HeJ mice infected with *B. burgdorferi* B31MI or with serum from hyperimmunized mice (1:1000 dilution). Ab binding was detected using horseradish peroxidase (HRP)-conjugated secondary Ab (1:40,000 dilution; Pierce) and SuperSignal West Pico chemiluminescence substrate (Pierce). After initial immunoblot analyses localized epitopes within residues 111–150 and 221–273, ~20 aa subfragments of these regions were generated with 10 aa overlaps and assessed by immunoblotting as detailed above.

### 2.4. Infection of mice and generation of antiserum

Six week old male C3H/HeJ mice (Jackson Laboratories) were infected by subcutaneous needle inoculation between the shoulder blades with 10^4^ spirochetes in a volume of 100 μl (PBS). Four weeks post infection, mice were sacrificed, tissue biopsies were collected and blood harvested by cardiac puncture. Infection was confirmed by cultivation of spirochetes from ear punch biopsies (2 mm) in BSK-H media (Sigma-Aldrich) containing antimicrobial cocktail (Sigma-Aldrich). To generate antiserum against the OspA_221–240_ epitope, a peptide corresponding to residues 221–240 was synthesized and conjugated to keyhole limpet hemocyanin (KLH) or unconjugated (UNC) on a fee for service basis (GenScript). Mice were immunized with 20 μg KLH-OspA_221–240_ or UNC-OspA_221–240_ peptide adsorbed to Imject Alum (Pierce) with boosts at 3, 6, and 9 weeks. Sera samples were collected by tail nick at weeks 0, 2, 4, and 8. At week 12, mice were sacrificed and terminal bleeds conducted by cardiac puncture.

### 2.5. Measurement of the IgG titer elicited by immunization with an OspA peptide spanning residues 221–240 (OspA_221–240_)

The IgG titer to KLH-OspA_221–240_ and UNC-OspA_221–240_ was determined by enzyme linked immunosorbent assays (ELISA). Ninety-six well plates (Costar 3590; Corning) were coated with 1 mg of full-length r-OspA per well in carbonate buffer (pH 9.6; overnight; 4 °C) and non-specific binding blocked (5% non-fat dry milk in PBS with 0.2% Tween-20 (PBSTM); 2 h). Serial dilutions of α-KLH-OspA_221–240_, UNC-OspA_221–240_ antiserum or α-KLH antiserum (1:50–1:109,350) were added to the wells (1 h; room temperature; in triplicate), the plates were washed 3 times (PBS-T) and HRP-conjugated goat α-mouse IgG (secondary Ab; 1:15,000) was added with ABTS serving as the chromogenic substrate. Absorbance was read at 405 nm in an ELISA plate reader (ELix 808; Biotek) during the linear phase of the reaction. Titers were calculated by fitting a logarithmic curve to the absorbance curve and calculating the inverse dilution corresponding to 1/3 of the maximum absorbance plateau as previously described [27]. Statistical analyses are detailed in figure legend 2.

### 2.6. Indirect immunofluorescence assays (IFA)

Surface exposure of the OspA_221–240_ epitope was assessed through IFA analyses. *B. burgdorferi* B31MI and B31 2E6 were grown to mid log phase, harvested and prepared for IFA using previously described-standard approaches [25]. Cells adhered to one set of slides were permeabilized with acetone and cells on a second set of slides were adhered by air drying. Non-specific binding was blocked with BSA-blocking buffer (1X PBS with 3% BSA and 0.2% Tween 20); and then the slides were screened with mouse preimmune serum, α-KLH-OspA_221–240_ antiserum, α-UNC-OspA_221–240_ antiserum, and mouse-α-OspA antiserum (see Fig. 3 for antiserum dilutions). AlexaFluor 568-conjugated goat-α-mouse IgG (1:500) was added for detection of Ab binding and the slides were assessed using an Olympus BX51 fluorescent microscope (fluorescein filter) or by dark-field microscopy. Images were digitally captured using an Olympus DP71 camera.

### 2.7. Measurement of the bactericidal activity of α-KLH-OspA_221–240_ Ab

Bactericidal activity of Ab induced by immunization with KLH-OspA_221–240_, UNC-OspA_221–240_ or KLH alone was determined as previously described [45] with minor modifications. Four μl of a mid-log phase culture of each strain was placed in 20 μl BSK-H complete media containing 20% α-KLH-OspA_221–240_ antiserum, 20% α-UNC-OspA_221–240_ antiserum, 20% α-KLH (negative control), or 20% α-full-length OspA with 20% complement-certified guinea pig serum (GPS; Complement Tech). Negative controls consisted of: (1) each strain incubated with the appropriate antiserum and 20% heat inactivated (HI) GPS, (2) cells in BSK-H with 20% GPS, and (3) cells in BSK-H alone. After incubation (37 °C; 18 h), viable cell numbers were determined by visual counting using dark-field microscopy. The average number of live and dead cells in ten representative 400x fields of view was determined for each sample and the data expressed as percent killing. All assays were performed in triplicate with two independent experimental replicates. Statistical significance was evaluated as indicated in the legend of Fig. 4.

## 3. Results

### 3.1. Identification of an OspA linear epitope

**Fig. 1.**
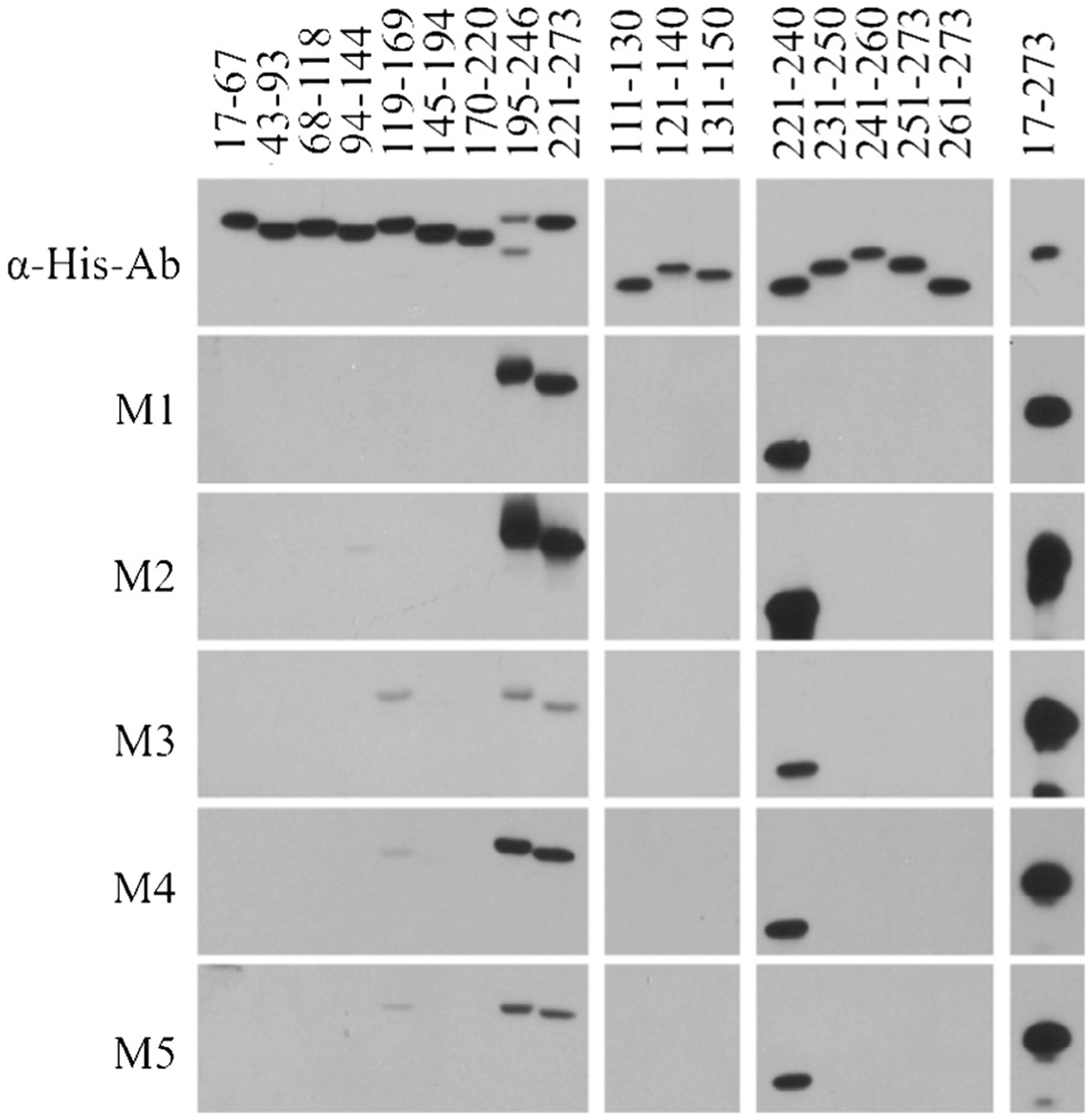
Identification of linear OspA epitopes. A series of OspA subfragments were generated with N-terminal S-tag fusions and purified. The recombinant proteins were separated by SDS-PAGE, transferred to membranes and screened with α-His tag Ab (top panel) or serum harvested from individual mice (M1 through M5) that were infected through needle inoculation with *B. burgdorferi* B31MI. Detection of Ab binding was performed as detailed in the text. The portion of OspA represented in each fragment is indicated above each lane.

Immunoblot screening of recombinant OspA subfragments with serum from mice infected with B31MI demonstrated the existence of an epitope within OspA residues 195–246 (OspA_195–246_) and or 221–273 (OspA_221–273_) (Fig. 1). Weak immunoreactivity with other OspA subfragments was observed in a subset of mice. The identification of epitopes contained within these subfragments was not pursued further. Immunoblot screening of overlapping subfragments of OspA_195–246_ localized the epitope within OspA_221–240_. It is important to note that since OspA is down regulated by spirochetes when transmitted to mammals by tick bite, in this study, mice were infected by needle inoculation using *in vitro* cultivated spirochetes in order to allow for the development of a robust Ab response to OspA.

### 3.2. Enzyme-linked immunosorbent assay (ELISA): Determination of α-OspA IgG titers induced by vaccination

**Fig. 2.**
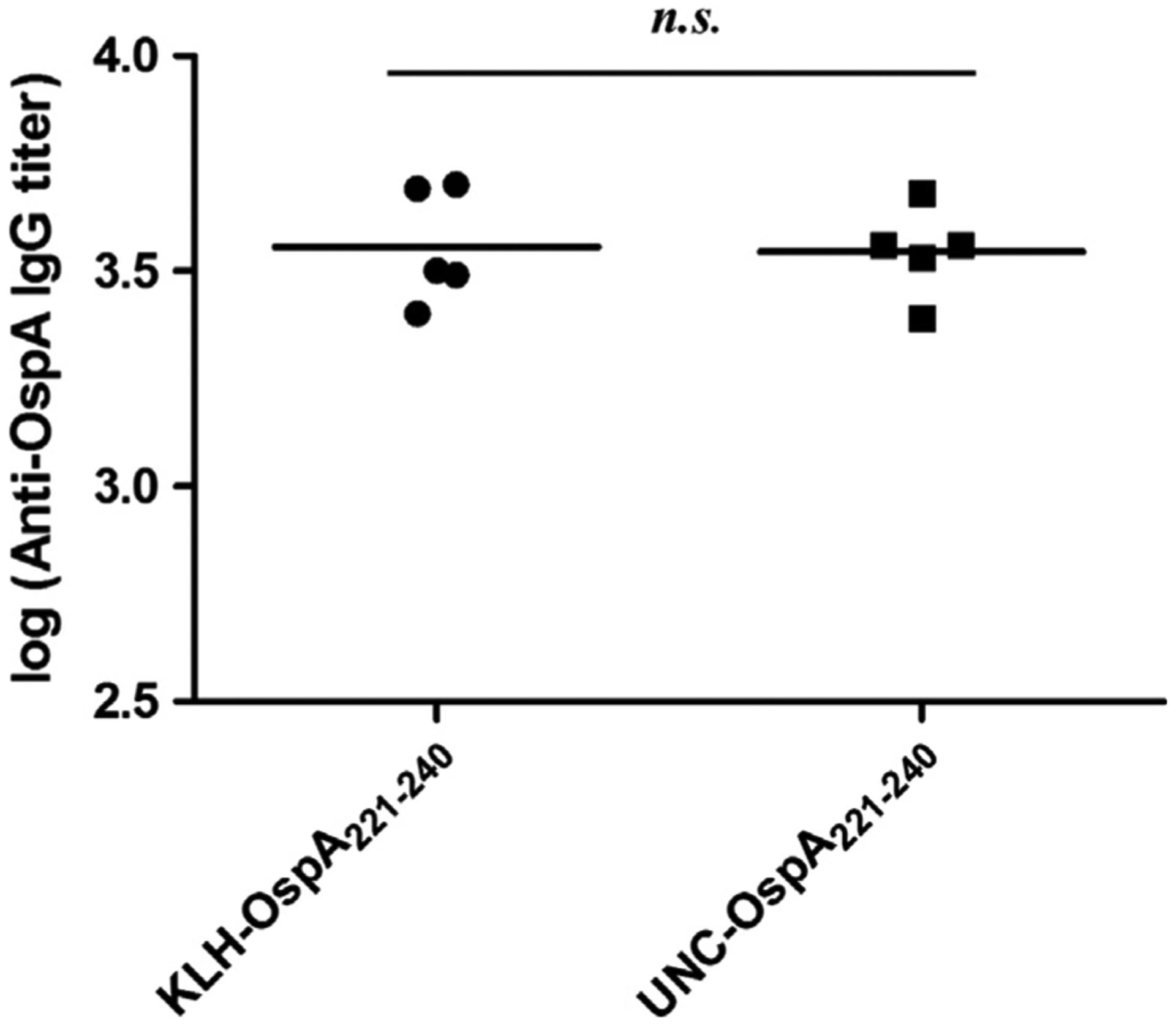
Analysis of the immunogenicity of KLH-conjugated and unconjugated OspA_221–240_ peptide. Unconjugated (UNC) and conjugated OspA_221–240_ peptide (KLH) were used to immunize mice and serum was harvested from each individual animal. The IgG titers elicited by vaccination with KLH-OspA_221–240_ or UNC-OspA_221–240_ were determined using full length OspA as the immobilized antigen. OspA screened with α-KLH antiserum served as a negative control (data not shown). Statistical significance was calculated using an unpaired, two-tailed student’s *t*-test (99.9% CI, p < 0.0001; *n.s.* indicates that the differences were not statistically significant). All methods were as detailed in the text.

IgG titers elicited in mice immunized with the KLH-conjugated and unconjugated (UNC) forms of OspA_221–240_ peptide or to KLH alone were determined by ELISA using full-length OspA (minus the leader peptide) as the immobilized antigen (Fig. 2). IgG titers to KLH-OspA_221–240_ and UNC-OspA_221–240_ were surprisingly similar with no statistical difference and varied little among mice indicating that titer was not dependent on KLH conjugation. No Ab to OspA was observed in serum from mice hyperimmunized with KLH alone (negative control). The data indicate that the OspA_221–240_ peptide is immunogenic.

### 3.3. The OspA_221–240_ epitope is accessible on the cell surface

**Fig. 3.**
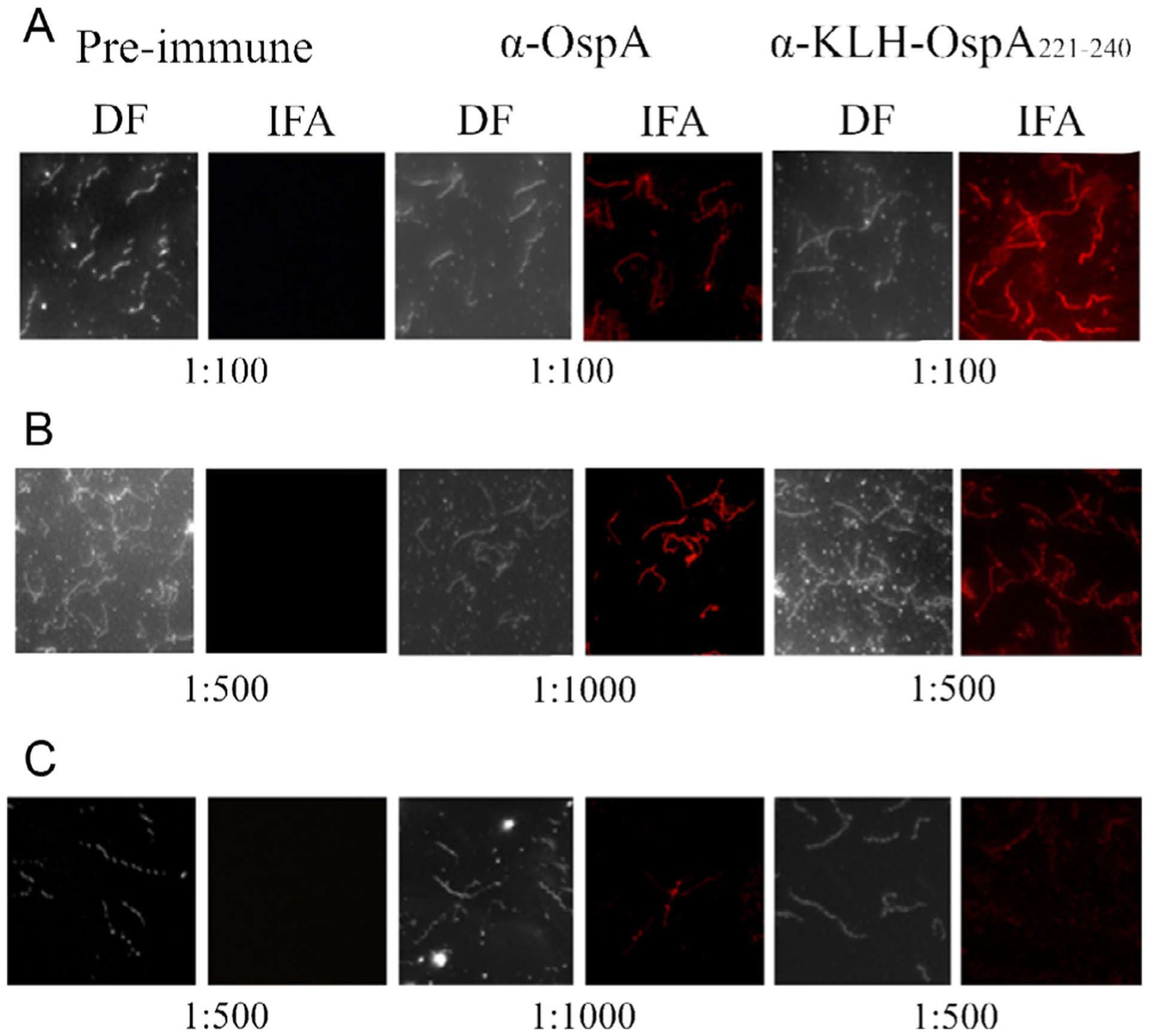
Demonstration that the OspA_221–240_ epitope is surface exposed on intact *B. burgdorferi* B31MI. Actively growing cultures of *B. burgdorferi* strains were spotted onto slides for IFA analyses as detailed in the text. Panel A, non-permeabilized (air dried) *B. burgdorferi* B31MI; Panel B, acetone permeabilized *B. burgdorferi* B31MI; Panel C, acetone permeabilized *B. burgdorferi* B31 2E6 (*ospA* gene inactivation mutant). Dark-field (DF) and fluorescent images are provided. The slides were screened with preimmune serum or antiserum as indicated along the top of the figure.

The ability of Ab elicited by hyper-immunization with KLH-OspA_221–240_ to bind to OspA in the context of an intact *B. burgdorferi* cell was assessed by IFA. Surface labeling of both intact cells and acetone permeabilized cells was observed indicating that the OspA_221–240_ epitope is naturally presented on the cell surface (Fig. 3). All cells in the population were labeled consistent with the universal and high level production of OspA by *B. burgdorferi* during *in vitro* cultivation [46]. As expected, no labeling was observed with cells exposed to pre-immune serum (negative control). Interestingly, *B. burgdorferi* B31 2E6, a strain that does not produce OspA [47], displayed significant surface labeling with α-OspA_221–240_ Ab. The probable basis for this observation is detailed below.

### 3.4. Ab targeting the OspA_221–240_ epitope has potent complement-dependent bactericidal activity

**Fig. 4.**
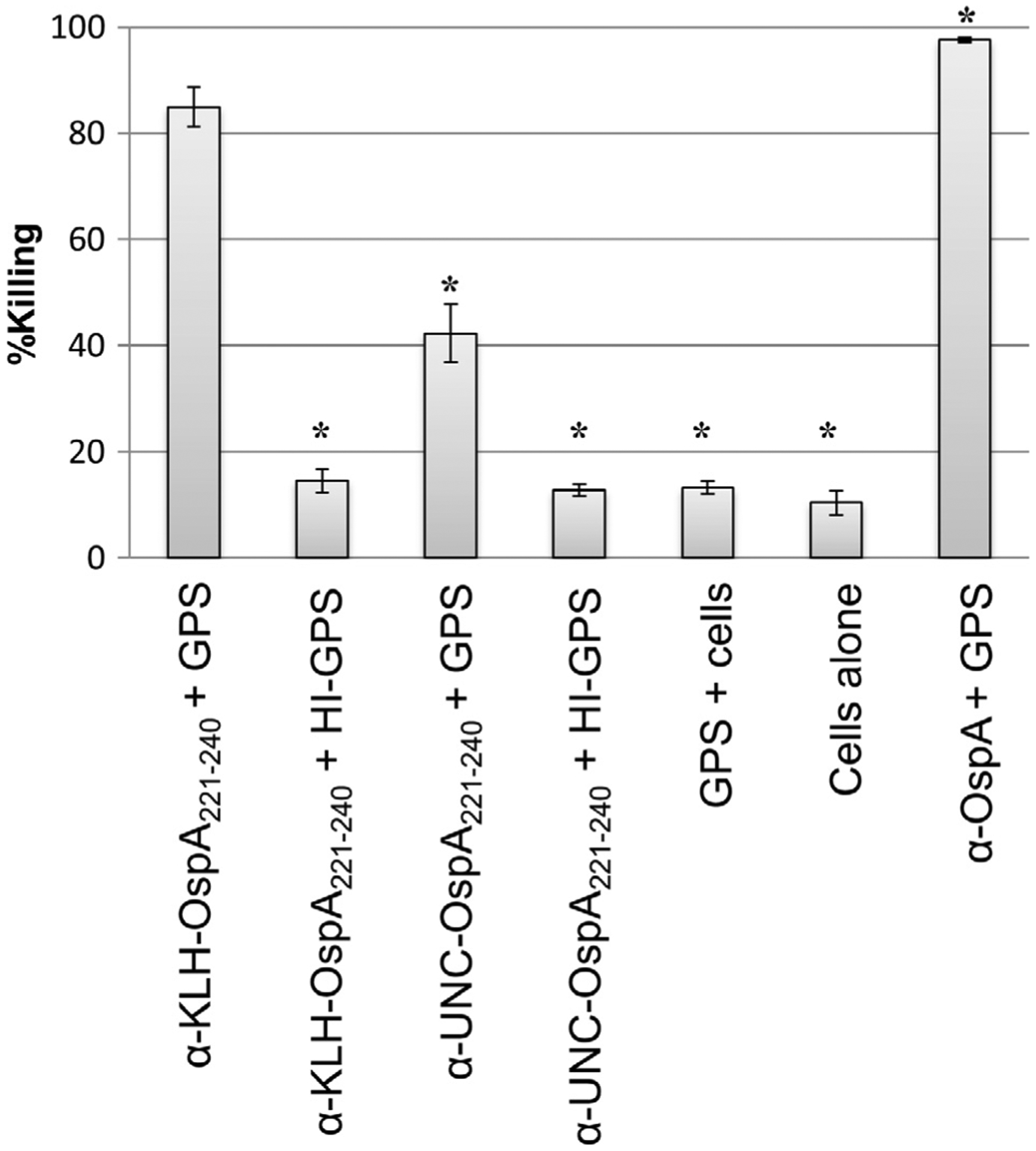
Ab to the OspA_221–240_ peptide is bactericidal. The bactericidal activity of α-OspA, α-OspA_221–240_ and α-KLH antisera against *B. burgdorferi* B31MI was assessed. Killing was quantified by counting of the number of live (motile and intact membrane) and dead cells (non-motile with membrane disruption) by dark-field microscopy (average of 10 fields of view). The data are expressed as percent killing. The One-Way ANOVA with post-hoc Dunnett’s comparison test was used to assess statistical significance (99.9% CI, p < 0.0001; significant differences in Ab mediated killing relative to that observed with α-OspA_221–240_ are indicated an asterisk). Abbreviations are as follows: guinea pig serum (GPS), heat inactivated (HI), keyhole limpet hemocyanin (KLH), and unconjugated (UNC).

**Fig. 5.**
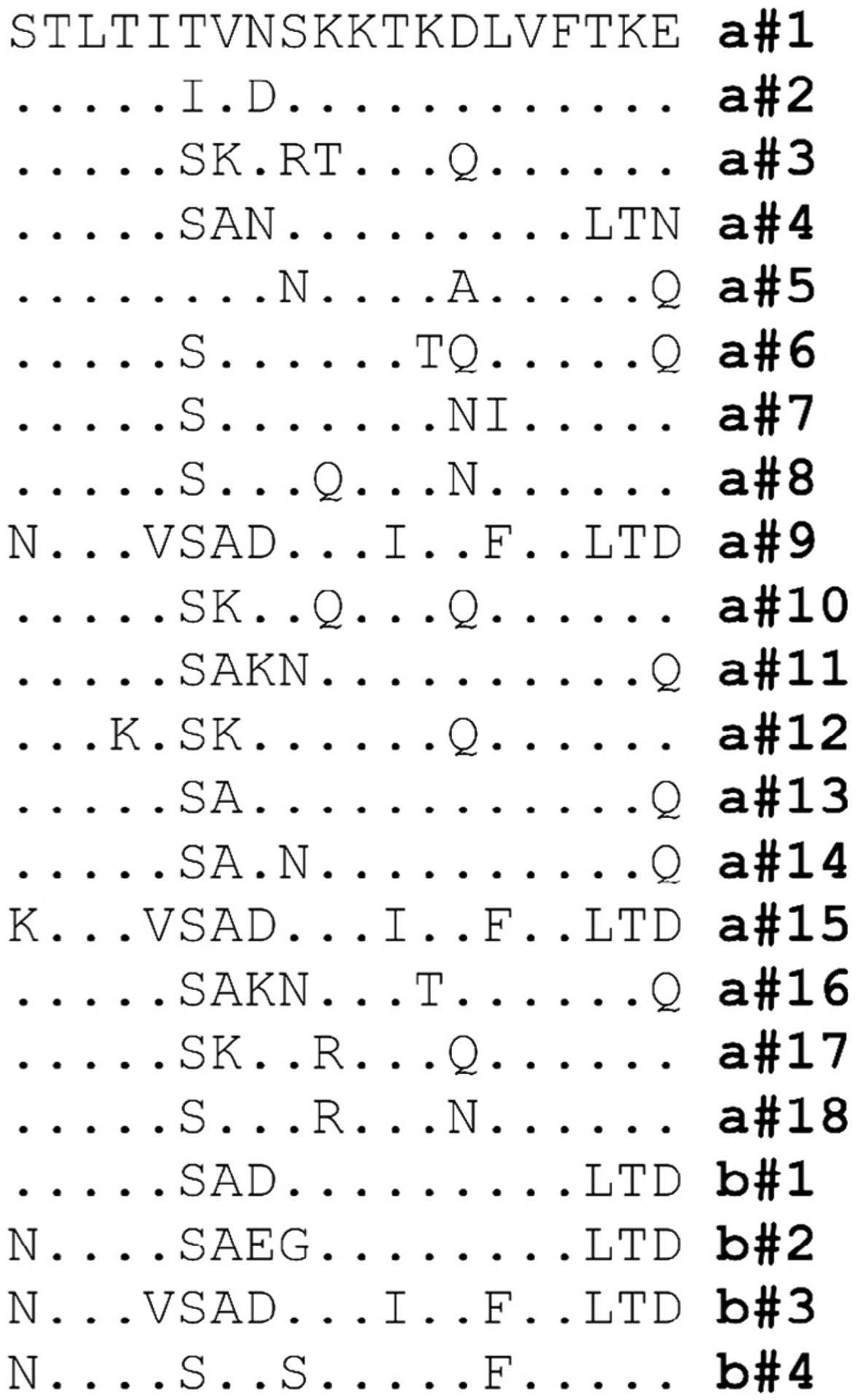
Alignment of OspA_221–240_ epitope variants. The *B. burgdorferi* B31 OspA sequence corresponding to residues 221 through 240 served as the query sequence for a BLAST search. The major variants of the epitope identified through BLAST are presented as a standard amino acid alignment. Note that since species-specific variants were not evident, the OspA_221–240_ and OspB_244–260_ variants were assigned letter/number designations (“a” for OspA and “b” for OspB followed by a number; a # 1, a # 2, b # 1, b # 2, etc.). Residues that are identical to the *B. burgdorferi* B31 OspA_221–240_ reference sequence are indicated by (.).

Incubation of *B. burgdorferi* B31MI with α-KLH-OspA_221–240_ or α-UNC-OspA_221–240_ and complement certified GPS resulted in 86% and 45% killing, respectively (Fig. 4). While KLH conjugation did not influence IgG titer, the proportion of bactericidal Ab appears to be higher in serum generated using the KLH-conjugated peptide. The bactericidal activity elicited by immunization with the KLH-OspA_221–240_ peptide was only slightly less than that observed with α-full-length OspA Ab. Killing of strains of other *Borrelia* species including *B. bavariensis* and *B. afzelii* by α-OspA_221–240_ Ab was also observed (data are summarized in Table 1). As detailed below, the lower level of killing of these strains is likely due to sequence diversity within the OspA_221–240_ epitope. Interestingly, significant killing of B31 2E6 (44.3%) was observed upon exposure to α-OspA_221–240_ antiserum and GPS. As discussed in detail below, the OspB protein possesses a sequence with 82.5% aa identity to the OspA_221–240_. It is possible that killing of the B31 2E6 strain resulted from targeting of the related sequence in OspB. When *ospB* was selectively inactivated in an *ospA*+ background, 100% killing was observed after incubation of cells with α-OspA_221–240_ Ab. The data suggest that both OspA and OspB are targets for α-OspA_221–240_ Ab. In all bactericidal assays described above, Ab mediated killing proved to be complement dependent as no significant killing of any strain was observed when HI-GPS was used as the complement source. Fig. 5 presents an alignment of different OspA_221–240_ sequences as well the related sequences found in OspB.

## 4. Discussion

Efforts in our laboratory to develop a next generation human Lyme disease vaccine and improved veterinary vaccines have focused on the development of novel chimeric OspC epitope based proteins referred to as “chimeritopes” [25–27,38,39,48,49]. It is our hypothesis that an OspA-OspC chimeritope will elicit comprehensive broad protection against diverse Lyme disease strains and protect through dual synergistic mechanisms; blocking or attenuation of spirochete transmission from ticks to mammals and killing of spirochetes that successfully transmit into mammals. In this study, we sought to identify OspA linear epitopes that could be incorporated into OspC based chimeritopes.

Here, we identify and characterize an OspA linear epitope present within residues 221–240 (OspA_221–240_). Ab elicited by immunization of mice with KLH-OspA_221–240_ peptide surface labeled *B. burgdorferi* cells indicating that the epitope is surface exposed on the protein. Ab also displayed potent complement dependent-bactericidal activity that was nearly equivalent to that of Ab raised against the full-length protein. This finding raises the possibility that the OspA_221–240_ epitope may be the dominant epitope responsible for the potent killing activity of Ab that develops in response to vaccination with full-length OspA.

The potential of α-OspA_221–240_ Ab to kill diverse species or strains of the Lyme disease spirochetes was tested by incubation of α-OspA_221–240_ antiserum from hyperimmunized mice with cultures of *B. burgdorferi* DRI40*, B. bavariensis* PbaeII and PHoe and *B. afzelii* J1. *B. burgdorferi* strain DRI40 was killed at a level equivalent to that of isolate B31. Low level bactericidal activity, relative to that observed with *B. burgdorferi* isolates, was noted with *B. bavariensis* and *B. afzelii* isolates. This data suggest that the immunodominant epitopes of OspA reside with variable domains of the protein and not conserved domains. Analysis of the expansive data set of OspA sequences revealed sequence variation within the OspA_221–240_ epitope among isolates that may account for the varying levels of killing. BLAST analyses using the OspA_221–240_ epitope sequence as the query identified several distinct OspA_221–240_ variants (see Fig. 5). OspA_221–240_ is conserved among OspA sequences originating from N. American isolates which are universally of OspA type a#1 but variable among isolates originating from other parts of the world (a#2–a#15). In an earlier study, a region immediately adjacent to OspA_221–240_, (OspA residues 247–256; QYDSNGTKLE) was demonstrated to bind the OspA mAb, B3G11 [50]. B3G11 also displayed complement dependent-bactericidal activity against *B. burgdorferi* B31 but not against isolates of other species. A BLAST search revealed that sequences corresponding to OspA B31 strain residues 247–256 are conserved among many *B. burgdorferi* isolates but are divergent in other species of the Lyme disease spirochetes.

Surprisingly, α-OspA_221–240_ Ab displayed significant killing activity against B31 2E6. This strain is derived from B31 but it does not produce OspA due to an insertional inactivation within the *ospA* gene [47]. B31 2E6 was originally included in these analyses to serve as a negative control as we expected that this strain would not be killed by α-OspA Ab. BLAST analyses using B31 OspA_221–240_ as the query identified a sequence in OspB with 82.4 % aa identity over 16 residues (STLTISADSKKTKDLVFLTD; b#1) with 9 contiguous residues being identical to OspA_221–240_ (Fig. 5). The region of OspB that corresponds to the 16 aa segment of OspA_221–240_ maps to residues 244 through 260 of *B. burgdorferi* B31 OspB [11]). A BLAST search using the OspB_244–260_ as the query revealed this sequence to be conserved among *B. burgdorferi* OspB sequences with varying degrees of divergence among *B. garinii, B. bavariensis, B. afzelii* and other Lyme disease spirochete species (Fig. 5). Consistent with the decreased killing of *B. bavariensis* and *B. afzelii,* residues that differ from those in *B. burgdorferi* are centrally located within OspB_244–260_. The positioning of these mismatches would likely disrupt epitopes and decrease Ab binding. Based on the similarities between *B. burgdorferi*, OspA_221–240_ and OspB_244–260_, it appears that most Lyme disease isolates possess two probable targets for α-OspA_221–240_ Ab as well as for α-OspA_full length_. This likely explains the potent bactericidal activity elicited by the OspA_221–240_ epitope and α-OspA_full length_. Interestingly, the binding site of the bactericidal OspB mAb, H6831, has been localized to the region in and around residue 253 of OspB [51]. Collectively, data provided by Sadziene et al. and in this report indicate that the OspB_244–260_ region of OspB is presented on the surface of the protein and cell.

Prior to this study, efforts to develop modified OspA proteins for human vaccine applications have largely assumed that OspA protective epitopes are conformational, discontinuous and located within the C-terminal half of the protein [52,53]. One study reported that α-OspA Ab that can surface label cells and neutralize *B. burgdorferi* can only be generated using full-length OspA [54]. Subfragments of OspA spanning different regions within residues 76–219 failed to induce productive Ab responses. Two of these fragments, fragment 8 (residues 133–273) and fragment 9 (residues 183–273), include the OspA_221–240_ epitope. A possible explanation for the inability of fragments 8 and 9 to induce productive Ab is that the potentially non-native structure of these subfragments prevents surface presentation of OspA_221–240_. Consistent with this, Koide et al. reported that a recombinant lipidated subfragment of OspA spanning residues 130–273 was conformationally unstable [53]. However, substitution of aa residues R139, E160 and K189 with M, Y and M, respectively, increased the stability of the lipidated fragment and elicited Ab in hyperimmunized mice that blocked transmission of *B. burgdorferi* from ticks. Here, we demonstrate that lipidation and maintenance of native OspA conformation are not required to stimulate productive Ab responses as bactericidal activity was obtained using the OspA_221–240_ peptide.

In another study supported by Baxter, a multivalent OspA based vaccine was assessed for immunogenicity and safety in a randomized double blinded phase I/II trail [36]. The multivalent vaccine was designed to provide broader protection than that afforded by previous vaccines that utilized a single OspA variant of N. American origin. While the vaccine was immunogenic and displayed a good safety profile, an intensive booster schedule was proposed. Specifically, three doses of alum adjuvanted vaccine were delivered 28 days apart followed by booster doses at 6, 9, or 12 months after the first dose. The study did not investigate *in vitro* correlates of protection such as the bactericidal activity of vaccine induced Ab against diverse Lyme disease spirochete strains.

With the identification of the OspA_221–240_ epitope, we will in future studies seek to develop OspC-OspA based chimeritopes consisting of multiple variants of the OspC L5, OspC H5, OspA_221–240_ and OspB_244–260_ epitopes. A single chimeritope protein consisting of epitopes derived from different Lyme disease spirochete proteins and diverse variants is innovative and has the potential to protect through multiple synergistic mechanisms with broad protective range. In addition, the inclusion of epitopes derived from distinctly different proteins will lead to Ab responses that can attack multiple targets on the *Borrelia* surface, effectively amplifying killing activity.

## Figures and Tables

**Table 1:** Strain description and summary of bactericidal assays.

Species and strain description/OspA designation	% Killing with α-OspA_221–240_	% Killing with α-OspA_full length_	% Killing with α-KLH
*B. burgdorferi* B31MI (OspA type a#1): clonal population derived from isolate B31 that was isolated from an Ixodes scapularis tick (USA) [55]	84.9 ± 3.73	97.6 ± 0.46	0
*B. burgdorferi* 2E6 (OspA type a#1): ospA gene inactivation mutant derived from the B31 clone 5A3 [47]	44.3 ± 1.8	100 ± 0	ND
*B. burgdorferi* 7A (OspA type a#1): ospB gene inactivation mutant derived from the B31 clone 5A3 [47]	100 ± 0	100 ± 0	ND
*B. burgdorferi* DRI40 h (OspA type a#1): clonal population derived from strain DRI40 that was recovered from a purpose bred beagle infected with field collected ticks (USA) [49]	84.0 ± 1.8	98.1 ± 1.7	ND
*B. bavariensis* PbaeII (OspA type a#7): cerebrospinal fluid of a human Lyme disease patient (Germany) [56]	70.0 ± 2.0	97.4 ± 2.1	ND
*B. bavariensis* Phoe (OspA type a#7): cerebrospinal fluid of a human Lyme disease patient (Germany) [56]	70.9 ± 3.9	100 ± 0	ND
*B. afzelii* J1 (OspA type #6): I. persulacatus tick (Japan) [57]	36.8 ± 4.3	98.3 ± 0.4	ND
*B. afzelii* B023 (OspA type unknown): skin biopsy from an erythema migrans lesion from a patient (Germany) [58]	27.9 ± 2.2	100 ± 0	ND
*B. afzelii* DK26: skin biopsy from an erythema migrans lesion from a patient (Denmark) [59]	15.3 ± 4.8	98.8 ± 1.1	ND

ND: not determined.

**Table 2:** Oligonucleotide primers.^a^

Primer designation	Primer sequence (5′–3′)	Region encoded by amplicon^b^
OspA 17 LIC (+)	**GACGACGACAAGATT**TGTAAGCAAAATGTTAGCAGC	17–67
OspA 67 LIC (−)	**GAGGAGAAGCCCGGTTTA**AGAAGTTCCTTTAAGCTCAAGCT	
OspA 43 LIC (+)	**GACGACGACAAGATT**AGCAAAGAAAAAAACAAAGACG	43–93
OspA 93 LIC (−)	**GAGGAGAAGCCCGGTTTA**ATCGTCAGAAATTGTTAATTTTACTT	
OspA 68 LIC (+)	**GACGACGACAAGATT**GATAAAAACAATGGATCTGGAGTAC	68–118
OspA 118 LIC (−)	**GAGGAGAAGCCCGGTTTA**GTCTTTGGAAGTTACTTTTTTTGAT	
OspA 94 LIC (+)	**GACGACGACAAGATT**CTAGGTCAAACCACACTTGAAGT	94–144
OspA 144 LIC (−)	**GAGGAGAAGCCCGGTTTA**TCTGGTTCCGTCTGCTCTT	
OspA 119 LIC (+)	**GACGACGACAAGATT**AAGTCATCAACAGAAGAAAAATTC	119–169
OspA 169 LIC (−)	**GAGGAGAAGCCCGGTTTA**TCCTTCAAGAACATAGCCTTTTA	
OspA 145 LIC (+)	**GACGACGACAAGATT**CTTGAATACACAGGAATTAAAAGC	145–194
OspA 194 LIC (−)	**GAGGAGAAGCCCGGTTTA**AGATTTTGAAATATTTTTGCTTAAA	
OspA 170 LIC (+)	**GACGACGACAAGATT**ACTCTAACTGCTGAAAAAACAAC	170–220
OspA 220 LIC (−)	**GAGGAGAAGCCCGGTTTA**AGTGCCTGAATTCCAAGCT	
OspA 195 LIC (+)	**GACGACGACAAGATT**GGGGAAGTTTCAGTTGAACTTA	195–246
OspA 246 LIC (−)	**GAGGAGAAGCCCGGTTTA**TTGTACTGTAATTGTGTTTTCTTTTG	
OspA 221 LIC (+)	**GACGACGACAAGATT**TCAACTTTAACAATTACTGTAAACAGT	221–273
OspA 273 LIC (−)	**GAGGAGAAGCCCGGTTTA**TTTTAAAGCGTTTTTAATTTCATC	
OspA 111 LIC (+)	**GACGACGACAAGATT**TCAAAAAAAGTAACTTCCAAAGAC	111–130
OspA 130 LIC (−)	**GAGGAGAAGCCCGGTTTA**ACCTTTTTCATTGAATTTTTCTT	
OspA 121 LIC (+)	**GACGACGACAAGATT**TCAACAGAAGAAAAATTCAATGA	121–140
OspA 140 LIC (−)	**GAGGAGAAGCCCGGTTTA**TGCTCTTGTTATTATTTTTTCAGATA	
OspA 131 LIC (+)	**GACGACGACAAGATT**GAAGTATCTGAAAAAATAATAACAAGAG	131–150
OspA 150 LIC (−)	**GAGGAGAAGCCCGGTTTA**AATTCCTGTGTATTCAAGTCTGG	
OspA 221 LIC (+)	**GACGACGACAAGATT**TCAACTTTAACAATTACTGTAAACAGT	221–240
OspA 240 LIC (−)	**GAGGAGAAGCCCGGTTTA**TTCTTTTGTAAACACAAGGTCTTT	
OspA 231 LIC (+)	**GACGACGACAAGATT**AAAACTAAAGACCTTGTGTTTACAA	231–250
OspA 250 LIC (−)	**GAGGAGAAGCCCGGTTTA**TGAGTCGTATTGTTGTACTGTAATTG	
OspA 241 LIC (+)	**GACGACGACAAGATT**AACACAATTACAGTACAACAATACG	241–260
OspA 260 LIC (−)	**GAGGAGAAGCCCGGTTTA**AACTGCTGACCCCTCTAATTT	
OspA 251 LIC (+)	**GACGACGACAAGATT**AATGGCACCAAATTAGAGGG	251–273
OspA 273 LIC (−)	**GAGGAGAAGCCCGGTTTA**TTTTAAAGCGTTTTTAATTTCATC	
OspA 261 LIC (+)	**GACGACGACAAGATT**GAAATTACAAAACTTGATGAAATTAA	261–273

a Sequences included to allow for ligase independent cloning (LIC) are indicated in bold.

b Numbering is based on the *B. burgdorferi* B31 OspA sequence.
